# *Salvia miltiorrhiza* Extract and Individual Synthesized Component Derivatives Induce Activating-Transcription-Factor-3-Mediated Anti-Obesity Effects and Attenuate Obesity-Induced Metabolic Disorder by Suppressing C/EBPα in High-Fat-Induced Obese Mice

**DOI:** 10.3390/cells11061022

**Published:** 2022-03-17

**Authors:** Yueh-Lin Wu, Heng Lin, Hsiao-Fen Li, Ming-Jaw Don, Pei-Chih King, Hsi-Hsien Chen

**Affiliations:** 1 Graduate Institute of Clinical Medicine, College of Medicine, Taipei Medical University, Taipei 110, Taiwan; vincewu168@gmail.com (Y.-L.W.); linheng@tmu.edu.tw (H.L.); 2 Division of Nephrology, Department of Internal Medicine, Wei-Gong Memorial Hospital, Miaoli 350, Taiwan; 3 Institute of Molecular and Genomic Medicine, National Health Research Institutes, Miaoli 350, Taiwan; 4 Division of Nephrology, Department of Internal Medicine, Taipei Medical University Hospital, Taipei 110, Taiwan; 5 TMU Research Center of Urology and Kidney, Taipei Medical University, Taipei 110, Taiwan; 6 Department of Physiology, School of Medicine, College of Medicine, Taipei Medical University, Taipei 110, Taiwan; bubble0728@gmail.com (H.-F.L.); rawaking0420@gmail.com (P.-C.K.); 7 National Research Institute of Chinese Medicine, Taipei 112, Taiwan; mjdon@nricm.edu.tw; 8 Division of Nephrology, Department of Internal Medicine, School of Medicine, College of Medicine, Taipei Medical University, Taipei 110, Taiwan

**Keywords:** *Salvia miltiorrhiza*, ATF3, obesity, C/EBPα

## Abstract

Pharmacological studies indicate that *Salvia miltiorrhiza* extract (SME) can improve cardiac and blood vessel function. However, there is limited knowledge regarding the effects (exerted through epigenetic regulation) of SME and newly derived single compounds, with the exception of tanshinone IIA and IB, on obesity-induced metabolic disorders. In this study, we administered SME or dimethyl sulfoxide (DMSO) as controls to male C57BL/J6 mice after they were fed a high-fat diet (HFD) for 4 weeks. SME treatment significantly reduced body weight, fasting plasma glucose, triglyceride levels, insulin resistance, and adipogenesis/lipogenesis gene expression in treated mice compared with controls. Transcriptome array analysis revealed that the expression of numerous transcriptional factors, including activating transcription factor 3 (ATF3) and C/EBPα homologous protein (CHOP), was significantly higher in the SME group. ST32db, a novel synthetic derivative similar in structure to compounds from *S. miltiorrhiza* extract, ameliorates obesity and obesity-induced metabolic syndrome in HFD-fed wild-type mice but not ATF3^−/−^ mice. ST32db treatment of 3T3-L1 adipocytes suppresses lipogenesis/adipogenesis through the ATF3 pathway to directly inhibit C/EBPα expression and indirectly inhibit the CHOP pathway. Overall, ST32db, a single compound modified from *S. miltiorrhiza* extract, has anti-obesity effects through ATF3-mediated C/EBPα downregulation and the CHOP pathway. Thus, SME and ST32db may reduce obesity and diabetes in mice, indicating the potential of both SME and ST32db as therapeutic drugs for the treatment of obesity-induced metabolic syndrome.

## 1. Introduction

Mammals have two basic types of adipocytes, white and brown cells, which are named according to their anatomical presence in white adipose tissue (WAT) and brown adipose tissue (BAT), respectively [1]. White adipocytes primarily accumulate fat for energy storage, whereas brown adipocytes burn fat through the uncoupling of respiration via uncoupling protein 1 (UCP1)-mediated shiver-free thermogenesis [2,3]. Excessive food intake combined with lower energy expenditure, resulting in excess fat accumulation in WAT, is considered the main cause of obesity in modern life. In particular, visceral white fat expansion is highly correlated with metabolic diseases [4]. However, BAT plays a key role in thermogenesis in neonates and small mammals such as rodents. It converts the energy in lipids into heat to maintain body temperature early in life and during winter [1].

Moreover, BAT activation has been suggested to encourage energy expenditure, decrease adiposity, and prevent diet-induced obesity and obesity-induced pathologies such as diabetes [5,6]. However, although well-developed BAT can be found in infants, it gradually degenerates after childhood, and in human adults, varying degrees of retained BAT can only be found in the cervical region [7,8]. Recent studies on rodents and humans have revealed a second type of brown cell, known as beige or brite (brown-in-white) cells [9]. Beige/brite cells are thought to be another type of brown adipocyte that arises in WAT, and they differ from classical brown adipocytes in that they are inducible, because they are initially found in the traditional white fat depots of rodents exposed to cold or injected with β-adrenergic receptor agonists [10]. Thus, beige/brite cells have a thermogenic function similar to classical brown adipocytes, but may have the same development or origin as white adipocytes [1,3,10]. Evidence suggests that white adipocytes can be interconverted into an intermediate form of beige/brite adipocytes through transdifferentiation, a process known as “browning” [3]. Therefore, new strategies for addressing obesity have pivoted from lowering fat accumulation to promoting energy expenditure through the browning of WAT or the activation of BAT.

ATF3 is a member of the ATF/cAMP response element-binding (CREB) family of basic region leucine zipper transcription factors [11]. ATF3 gene expression is induced by various stress signals, including cytokines, chemokines, hypoxia, DNA damage, ER stress, and nutrient deprivation [11]. Although *atf3* has long been realized as a stress-inducible gene that promotes apoptosis [12], cumulative evidence suggests that ATF3 may play a role in general adaptive responses, such as environmental and nutritional alterations [13,14]. First, ATF3 expression is dramatically upregulated in the WAT of high-fat-diet (HFD)-fed obese mice [15], ob/ob mice, and db/db mice [16]. In contrast, a loss of ATF3 in *Drosophila* increases lipid accumulation in the fat body and causes an energy imbalance [17]. Notably, a pro-apoptotic role for ATF3 in beta cells was originally assumed, and ATF3^−/−^ mice were predicted to be protected in a T2D model of HFD-induced diabetes. However, HFD-fed ATF3^−/−^ mice performed worse than WT mice in glucose tolerance tests and showed downregulated insulin gene transcription in their islets [18]. Furthermore, in pancreas- and hypothalamus-specific ATF3 knockout (PHT-ATF3-KO) mice, ATF3 was shown to play an important role in controlling glucose and energy metabolism by regulating agouti-related protein (Agrp), which increases food intake and reduces energy expenditure [14]. This evidence indicates that ATF3 may play a protective role in obesity and obesity-related metabolic diseases.

Indeed, emerging evidence and our recent study reveal that ATF3 regulates adipogenesis and reduces adiposity [19,20,21]. HFD-fed ATF3^−/−^ mice had aggravated weight gain, developed impaired glucose metabolism, and had hyperlipidemia compared with littermate wild-type (WT) control mice [19,20]. In contrast, adeno-associated virus 8 (AAV8)-virus-mediated ATF3 restoration reversed the obesity exacerbation and metabolic disorders in HFD-fed ATF3^−/−^ mice [19]. In addition, the overexpression of ATF3 in 3T3-L1 adipocytes suppressed adipogenesis/lipogenesis [19,22] and promoted browning [19]. Notably, a significant positive correlation was found between chalcone-mediated ATF3 induction level and lipid accumulation in C3H10T1/2 adipocytes [20]. Moreover, the literature shows that sulfuretin, a major flavonoid from *Toxicodendron vernicifluum*, effectively prevents HFD-induced obesity via ATF3 induction [23]. These data highlight the role of ATF3 in adipogenesis/lipogenesis and browning, and further suggest the potential application of natural or chemical ATF3 inducers for the treatment of obesity and its related metabolic diseases.

*Salvia miltiorrhiza* (or *S. miltiorrhiza* radix), which belongs to the family Labiatae, is a well-known traditional Chinese herb used to treat various diseases due to its excellent medicinal properties in improving cardiovascular health and inhibiting platelet aggregation [24]. Numerous chemical compounds have been isolated from *S. miltiorrhiza*. Currently, >50 water-soluble components and >30 liposoluble components isolated from *S. miltiorrhiza* are available [25]. The water-soluble components are mainly phenolic acid compounds such as danshensu (salvianolic acid A), salvianolic acid B, protocatechuic aldehyde, caffeic acid, and rosmarinic acid, and the liposoluble components are mainly conjugated quinones and ketones such as tanshinone I, tanshinone IIA, and cryptotanshinone [25]. Modern pharmacological analyses have revealed that most of the water-soluble components of *S. miltiorrhiza* have antioxidant, anticoagulation, and anti-myocardial ischemia effects, which are closely related to traditional effects such as blood activation and blood stasis removal [24,26]. In contrast, the liposoluble components, mainly tanshinones, have antibacterial, anti-inflammatory, myocardial protective, and endothelial cell protective properties [24,27]. However, to the best of our knowledge, almost no research exists on the anti-obesity effects of *S. miltiorrhiza* extract (SME), and few individual liposoluble components of *S. miltiorrhiza*, apart from tanshinone I and tanshinone IIA, have been discussed regarding their anti-obesity effects [28,29,30].

Notably, in our previous study, we found that *S. miltiorrhiza*-related compounds and their chemically synthesized derivatives ranked at the top of the list when we screened ATF3 inducers from the modified Chinese herb single-compound library at the National Research Institute of Chinese Medicine [19]. ST32db, a lipid-soluble chemically synthesized derivative with structural similarity to components of salvia, is among the top five candidates for ATF3 induction. Therefore, we hypothesized that ST32db might have an excellent anti-obesity effect via its strong ATF3 inducibility. Although Ku et al. recently reported that ST32db could induce browning by suppressing the p38 pathway in vivo [21], most known browning inducers upregulate the p38 pathway and then promote UCP1 production, such as cardiac natriuretic peptides or the T3 hormone [31]. Thus, we prefer ST32db to induce ATF3 upregulation to affect adipogenesis, which deserves further study. The study described here investigated the anti-obesity effects of *S. miltiorrhiza* components and ST32db, a novel modified liposoluble compound from *S. miltiorrhiza* components, on mice with HFD-induced obesity and explored the corresponding molecular mechanism, which occurs via the ATF3-mediated signaling pathway.

## 2. Materials and Methods

### 2.1. Animal Studies

In this study, 6-week-old male C57BL/6J and ATF3^−/−^ mice were used. The ATF3^−/−^ mice were kindly provided by Tsonwin Hai [32] and were backcrossed into C57BL/6J mice for at least 7 generations before the experiments in our previous study [19]. We established an obese mouse model through the consumption of a high-fat diet (D12451, with 45% of calories derived from fat; Research Diets, New Brunswick, NJ, USA) to C57BL/6J and ATF3^−/−^ mice, as previously described [19,33]. To monitor the effects of ST32db and *S. miltiorrhiza* treatments, 8-week-old WT and ATF3^−/−^ mice were fed an HFD with or without intraperitoneal (i.p.) ST32db (75 mg/kg/week) or *S. miltiorrhiza* (6 and 30 mg/kg/week) treatment. We measured body weight and food intake every week throughout the experimental period. Body composition was measured using a time-domain nuclear magnetic resonance (NMR) analyzer (Minispec LF-50; Bruker Optics). Insulin sensitivity and glucose tolerance tests were performed at the end of the treatment period. Then, the mice were sacrificed, and tissue and blood samples were collected. All procedures were performed according to the protocols of the Institutional Animal Care and Utilization Committee of Taipei Medical University, Taipei, Taiwan.

### 2.2. Glucose and Insulin Tolerance Tests

Measurements of plasma glucose levels in the collected blood samples were performed using a commercially available glucose meter (MultiSure GK Blood Glucose and Ketone Meter; ApExBio, Hsinchu, Taiwan). To conduct the glucose tolerance tests, mice were fasted overnight for 16 h, after which they received an i.p. injection of glucose (2 g/kg body weight) in saline. Plasma glucose levels in blood samples collected from the tail vein were measured 0, 15, 30, 60, 90, and 120 min after glucose injection. To perform the insulin tolerance tests, mice were fasted for 6 h, after which they received an i.p. injection of insulin (1 U/kg body weight) in saline. Plasma glucose levels in blood samples collected from the tail vein were measured 0, 15, 30, 60, 90, and 120 min after insulin injection.

### 2.3. Measurement of Biochemical Parameters

Blood samples (500 µL) were collected from the tail vein and centrifuged (6000× *g* for 3 min) to separate serum from cells. Serum biochemical parameters were measured within 24 h. The levels of serum blood urea nitrogen (BUN), creatinine, glucose, triglycerides (TG), glutamic oxaloacetic transaminase (GOT), and glutamate pyruvate transaminase (GPT) were measured using a Spotchem EZ SP 4430 analyzer (ARKRAY, Kyoto, Japan).

### 2.4. Histology, Adipocyte Size Measurement, and Adipocyte Number Estimation

For hematoxylin and eosin staining, adipose tissues were dissected and fixed in 4% paraformaldehyde overnight at 4 °C, and then fixed in paraffin before sectioning and staining. Stained WAT sections were analyzed using ImageJ. To determine the adipocyte size, 20 consecutive fat cells of the gonadal fat pad of mice were selected for measurements. The number of adipocytes in the gonadal fat pad was calculated as the fat pad volume divided by the average fat cell volume.

### 2.5. TF Activation Profiling Analysis

Each array assay was performed following the procedure described in the TF activation profiling plate array kit user manual (Signosis, Inc., Santa Clara, CA, USA). First, 15 μg of nuclear extract was incubated with a biotin-labeled probe mix at room temperature for 30 min. The activated TFs were bound to the corresponding DNA-binding probes. After protein/DNA complexes were isolated from unbound probes, the bound probes were eluted and hybridized on a plate precoated with captured oligos. The captured biotin-labeled probes were then detected using streptavidin–HRP and subsequently measured using a chemiluminescent plate reader (Fluoroskan Ascent FL, Thermo Fisher, Waltham, MA, USA).

### 2.6. Cell Culture

For the cell culture in this study, 3T3-L1 cells were maintained in Dulbecco’s modified Eagle’s medium supplemented with 10% calf serum, 100 U/mL penicillin, and 0.1 mg/mL streptomycin. For 3T3-L1 differentiation experiments, 2 days after the 3T3-L1 cells reached confluence (referred to as day 0), they were differentiated in culture medium supplemented with 5 μg/mL insulin, 0.5 mM 3-isobutyl-1-methylxanthine, and 1 μM dexamethasone for 2 days, and then maintained in culture medium supplemented with 5 μg/mL insulin. To analyze the effect of ST32db on cell differentiation, 3T3-L1 cells were treated with 60 μM ST32db for 8 days of differentiation. To analyze the effect of ATF3 depletion, 3T3-L1 cells were seeded in a 6-well plate at a density of 5 × 10^4^ cells per well and transiently transfected with pLKO.1-Emp as a control and shRNA-ATF3 using Lipofectamine™ 2000 (Thermo Fisher Scientific, Waltham, MA, USA). After 48 h of incubation, cells were treated with 60 μM ST32db for 6 h.

### 2.7. Oil Red O Staining

After 8 days of 3T3-L1 preadipocyte differentiation, differentiated adipocytes were washed twice with phosphate-buffered saline and fixed for 1 h in 10% formalin. The cells were then stained with Oil Red O working solution for 30 min. They were then washed 4 times with distilled water before microscopy analyses. The stained Oil Red O was eluted with 100% isopropanol (*v*/*v*) and quantified by measuring the optical absorbance at 500 nm.

### 2.8. Real-Time Polymerase Chain Reaction

Total RNA was extracted from cultured cells or adipose tissues using TRIzol reagent (Invitrogen, Thermo Fisher Scientific, Waltham, MA, USA), and RNA was reverse-transcribed to cDNA using the iScript cDNA Synthesis Kit (Bio-Rad, laboratories, Hercules, CA, USA). Real-time quantitative polymerase chain reaction (qPCR) analysis was performed using an ABI StepOnePlus Real-Time PCR System (Applied Biosystems, Grand Island, NY, USA) with SYBR green (Bio-Rad). The primer sequences used for qPCR are presented in Table S1.

### 2.9. Chromatin Immunoprecipitation Assay

The 3T3-L1 cells were fixed in 1% formaldehyde, and chromatin immunoprecipitation (ChIP) was performed following the Upstate protocol (Millipore, Merck Ltd., Taipei, Taiwan). Chromatin was immunoprecipitated using the anti-ATF3 antibody (Abcam, Cambridge, UK, ab254268). Purified DNA was detected using standard real-time PCR analysis. The corresponding primers are presented in Table S1.

### 2.10. Statistical Analysis

Values are expressed herein as mean ± standard error of the mean from at least 3 experiments. Analysis of variance followed by Tukey’s test was used to determine statistical significance in the in vivo experiments. *p* < 0.05 was considered to indicate statistically significant differences.

## 3. Results

### 3.1. SME Treatment Ameliorates HFD-Induced Metabolic Dyshomeostasis

**Figure 1:**
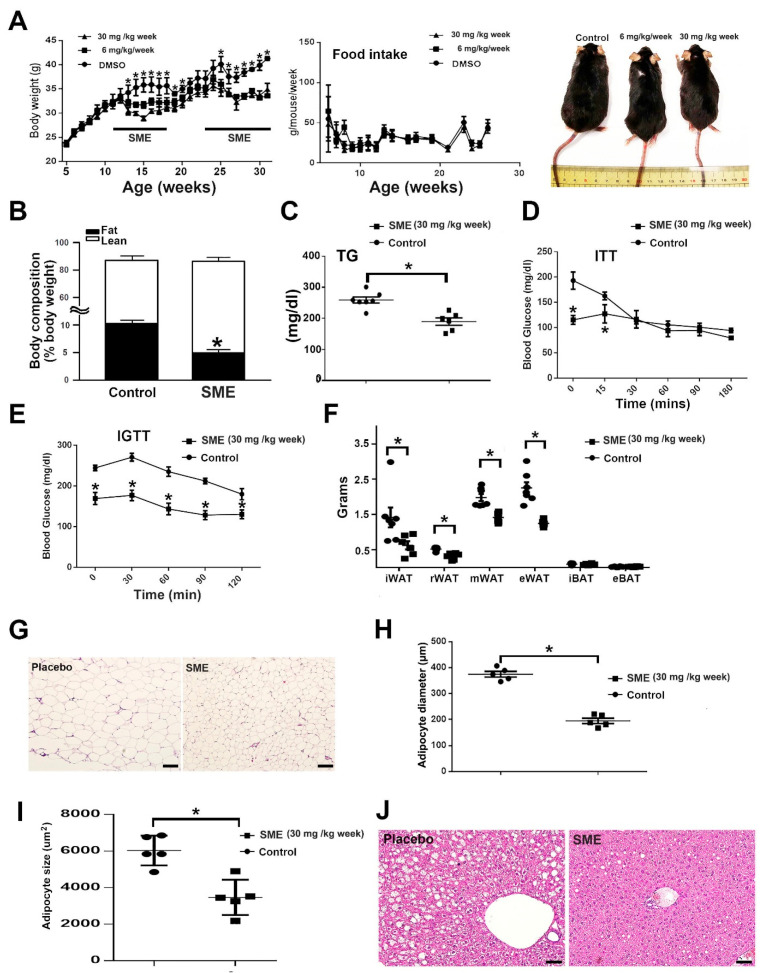
Results of treatment with or without *Salvia miltiorrhiza* extract (SME) in wild-type (WT) mice with high-fat-diet (HFD)-induced obesity and metabolic dysfunction. (**A**) Body weight, food intake measurements, and body imaging for placebo and SME treatment groups. (**B**) Body composition analysis. (**C**) Serum triglyceride (TG) levels. (**D**) Insulin tolerance test results. (**E**) Glucose tolerance test results. (**F**) Weights of inguinal WAT (iWAT), retroperitoneal WAT (rWAT), mesenteric WAT (mWAT), epididymal WAT (eWAT), interscapular BAT (iBAT), and epididymal BAT (eBAT). (**G**) Representative photographs of hematoxylin and eosin (**H**,**E**) staining results for iWAT. (**H**) Adipocyte diameter (μm). (**I**) Adipocyte size (μm^2^). (**J**) Liver (**H**,**E**) staining results. Data are the mean ± SEM; * *p* < 0.05 compared with control. For (**A**–**F**), *n* = 7 per group; for (**H**,**I**), *n* = 5 per group. Scale bar = 50 μm.

We conducted experiments to examine whether SME treatment could modulate energy metabolism, body weight changes, and anti-obesity effects in HFD-fed mice. HFD-fed WT mice were subjected to SME treatment at 30 or 6 mg/kg/week, and their body weights were compared with those of the placebo group. As illustrated in Figure 1A, the results revealed that the mice which received SME treatment at 30 mg/kg/week achieved a significantly reduced body weight compared with the placebo group at the second week after treatment. In contrast, the mice that received SME treatment at 6 mg/kg/week achieved a significantly reduced body weight compared with the placebo group at the third week after treatment, indicating that treatment at a higher dose was more effective in promoting weight reduction. However, no significant difference in food intake was observed between the treated and untreated groups of mice (Figure 1A). Due to the surprising effect of SME, we performed a re-challenge test, whereby we stopped SME injection while maintaining an HFD for 1 month and then re-administered SME. For the first 2 weeks after suspending SME, the treated mice did not regain their body weight quickly (Figure 1A).

Further assessments of SME treatment 3 weeks after re-administration still showed an anti-obesity effect similar to the results with the first administration (Figure 1A). The SME-treated mice exhibited reduced body fat percentages, serum TG levels (Figure 1B,C), insulin resistance and glucose intolerance (Figure 1D,E), WAT depot weight (Figure 1F), white adipocyte cell diameter and size (Figure 1G–I), and hepatic steatosis (Figure 1J). According to these results, SME treatment suppressed white adipocyte accumulation but did not impact brown adipose tissue (Figure 1F). Additionally, it ameliorated obesity-induced metabolic disorders such as hyperglycemia, diabetes, and hepatic steatosis. Furthermore, SME treatment did not show liver/kidney toxicity, but might ameliorate non-alcoholic fatty liver disease (Figure S1).

### 3.2. SME Treatment Inhibits Adipogenesis and Ameliorates Lipogenesis/Lipolysis Imbalance in HFD-Fed Mice

**Figure 2:**
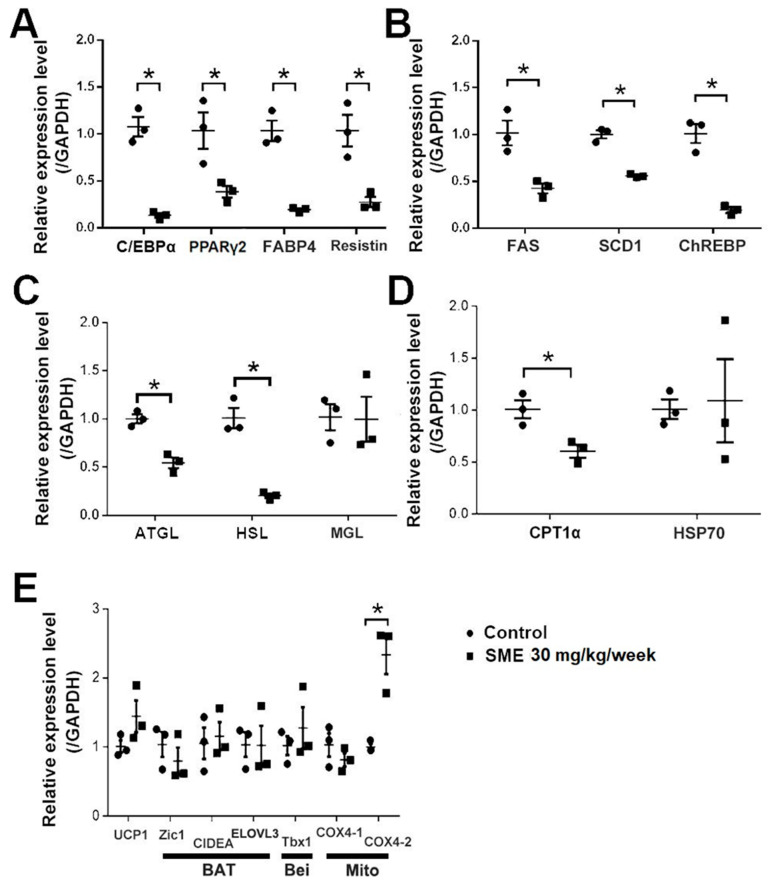
SME treatment ameliorates adipogenesis/lipolysis imbalance in HFD-fed mice. Effects of SME treatment on WT mice after 5 weeks of an HFD were analyzed. Expression of (**A**) adipogenic, (**B**) lipogenic, (**C**) lipolytic, (**D**) β-oxidation, and (**E**) brown (BAT), beige (Bei), and mitochondria (Mito) genes in inguinal WAT. Data are the mean ± SEM. (*n* = 3 per group); * *p* < 0.05 compared with control.

We further examined whether SME treatment could modulate body weight changes in mice, primarily by reducing adipocyte accumulation or impacting the BAT/WAT balance. In brief, WT mice were fed an HFD for 4 weeks, and then either administered SME 30 mg/kg/week or not while continuing an HFD. After 5 weeks of treatment, we sacrificed the mice and sampled inguinal WAT (iWAT) for further analysis. Accordingly, we analyzed the expression of adipogenesis and lipogenesis biomarkers, including C/EBPα, PPARγ2, fatty acid-binding protein 4 (*FABP4*), resistin, fatty acid synthase (*FAS*), stearoyl-CoA desaturase-1 (*SCD1*), and carbohydrate-responsive element-binding protein (*ChREBP*). The results revealed reduced expression levels of these biomarkers in the iWAT of SME-treated mice compared with untreated mice (Figure 2A,B); moreover, the expression levels of some lipolytic and β-oxidation biomarkers, including adipose triglyceride lipase (*ATGL*), hormone-sensitive lipase (*HSL*), and carnitine palmitoyltransferase 1α (*CPT1α*), were decreased in the treated mice. However, the levels of monoacylglycerol lipase (*MGL*) and 70 kDa heat shock protein (*Hsp70*) did not differ between SME-treated and untreated mice (Figure 2C,D). In contrast, the expression levels of specified biomarkers of BAT activation, WAT browning, and mitochondrial genes, including *UCP1*, zinc family member 1 (*Zic1*)*,* cell death activator CIDE-A (*CIDEA*)*,* elongation of very-long-chain fatty acid protein 3 (*ELOVL3),* T-box transcription factor 1 (*Tbx1*)*,* and cytochrome c oxidase subunit 4 isoform 1 (*COX4-1*)*,* but not cytochrome c oxidase subunit 4 isoform 2 (*COX4-2*), did not differ between SME-treated and untreated mice (Figure 2E). These results imply that SME treatment modulates body weight changes primarily through the inhibition of adipogenesis/lipogenesis, rather than WAT browning or BAT activation.

### 3.3. SME Treatment Changes the Expression of Numerous Transcriptional Factors in 3T3-L1 Adipocytes

**Figure 3:**
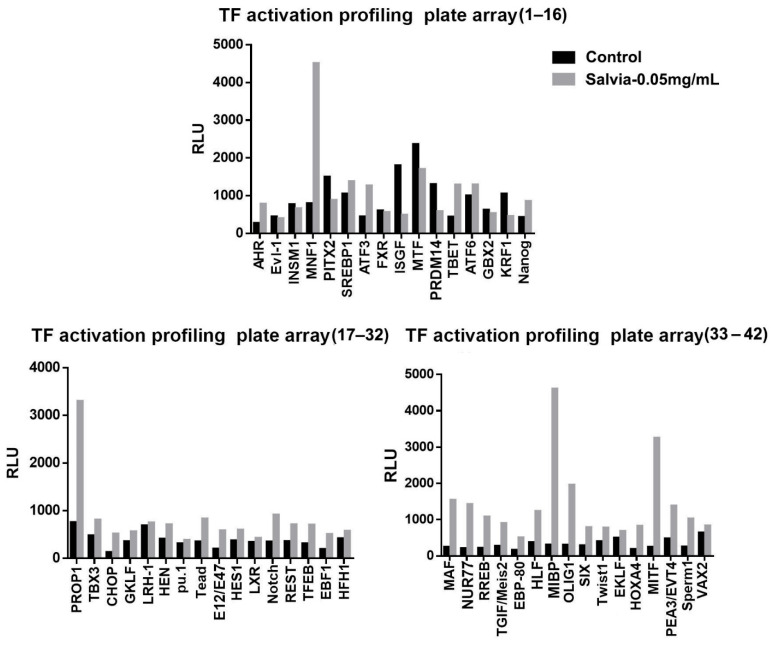
Transcriptomic analysis to identify candidate transcriptional factors in 3T3-L1 cells with or without SME treatment. AHR, aryl hydrocarbon receptor; ATF6, activating transcription factor 6; E12/E47, E2A immunoglobulin enhancer-binding factors; EBF1, early B-cell factor 1; EBP-80, emopamil binding protein 80; EKLF, erythroid Krüppel-like factor; Evl-1, myeloid transforming gene; FXR, nuclear receptor subfamily 1 group H member 4 (*Homo sapiens*); GBX2, gastrulation brain homeobox 2; GKLF, gut-enriched Krüppel-like factor; HFH1, HNF-3/fkh homolog gene; HES1, hes family bHLH transcription factor 1; HLF, hepatic leukemia factor; Sperm1, sperm-related protein; HOXA4, homeobox A4; INSM1, myeloid ecotropic viral integration site 1; ISGF, interferon regulatory factor; KRF1, keratinocyte-specific transcription factor; LXR, liver X receptor; MNF1, myocyte nuclear factor; MTF, myelin transcription factor; MAF, v-maf avian musculoaponeurotic fibrosarcoma oncogene; MIBP, c-myc intron binding protein 1; MITF, microphthalmia-associated transcription factor; Nanog, Nanog homeobox; HEN, nescient helix–loop–helix 1; Notch, Notch homolog, translocation-associated (*Drosophila*); NUR77, nerve growth factor IB (NGFIB); OLIG1, oligodendrocyte transcription factor 1; PEA3/EVT4, ETS translocation variant 4 (ETV4); PITX2, paired-like homeodomain 2; PRDM14, PRDI-BF1 and RIZ homology domain-containing protein 14; PROP1, paired-like homeobox 1; pu.1, ETS-domain transcription factor binding to purine-rich DNA sequence; REST/NRSF, repressor element 1 silencing transcription factor/neuron-restrictive silencing factor; RREB, Ras-responsive element binding protein 1; SREBP1, sterol regulatory element-binding protein 1; TBET, T-cell-specific T-box transcription factor; TBX3, T-box transcription factor; Tead, transcriptional enhancer factor; TEF-1, LRH-1 liver receptor homolog-1; TFEB, T-cell transcription factor EB; TGIF/Meis2, myeloid ecotropic viral integration site 1; Twist1, Twist basic helix–loop–helix transcription factor 1; VAX2, ventral anterior homeobox 2; SIX, SIX homeobox protein.

Previous studies examining transcriptomic changes in the pathogenesis of non-alcoholic fatty liver disease [34], obesity, type 2 diabetes mellitus [35], and hypertension [36] predominantly applied microarray-based techniques. To perform a comprehensive transcriptomic analysis in the present study, we used a commercial array (Signosis, Inc., catalog no. FA-1011) to identify candidate transcriptional factors regulated by SME. The array examined the responses of 48 genes to SME treatment, among which 37 were upregulated, 4 were downregulated, and 7 were unchanged (Figure 3). We found that most of the responsive genes were involved in cell division and chromosome partitioning, lipid metabolism, translation apparatus, and DNA replication and repair.

### 3.4. Synthesizing Individual Compounds as Candidate ATF3 Inducers

**Figure 4:**
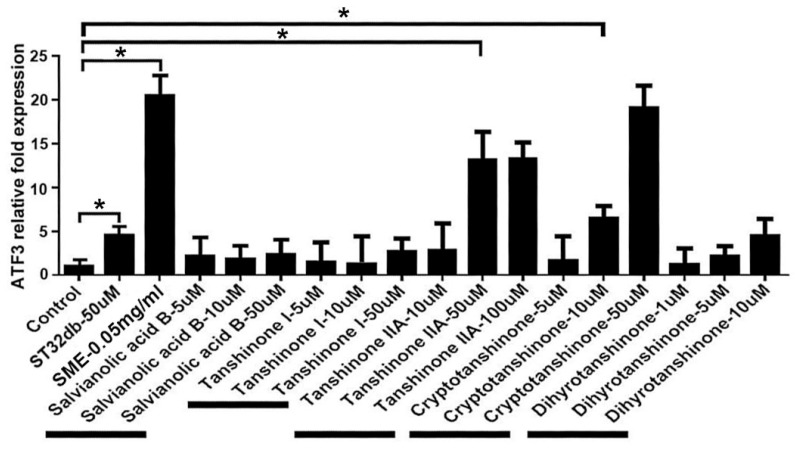
Upregulation of ATF3 expression was by purified single compounds derived from *S. miltiorrhiza*. 3T3-L1 cells were treated with single compounds as indicated, and real-time PCR analysis was conducted to determine ATF3 gene mRNA levels. Diagram represents the real-time PCR results from three independent experiments. Data are the mean ± SEM; * *p* < 0.05 compared with control.

The most abundant components from *S. miltiorrhiza* include salvianolic acid B, tanshinone I, tanshinone IIA, cryptotanshinone, and danshensu (salvianolic acid A), the basic chemical structures of which comprise four rings: naphthalene or tetrahydronaphthalene rings A and B, ortho- or para-quinone or lactone ring C, and furan or dihydrofuran ring D [37]. Our laboratory has synthesized more than 50 analogs of *Salvia miltiorrhiza* components. One of them, ST32da, an ATF3 inducer, has been shown to exert anti-obesity functions [19]. Coupled with the excellent anti-obesity effect of SME, we are interested in whether the major components in SME can also upregulate ATF3 expression. Therefore, we examined the ability of major components in SME to upregulate ATF3 in vitro. Notably, only tanshinone IIA and cryptotanshinone could induce ATF3 expression (Figure 4). Based on the basic structures of tanshinone IIA and cryptotanshinone, we further synthesized two compounds, ST32db and ST32C. To determine their purity, these compounds were assayed using mass spectrophotometry, high-performance liquid chromatography (HPLC), and NMR (Figure S2).

### 3.5. Individual Compounds as ATF3 Inducers Ameliorate HFD-Induced Metabolic Dyshomeostasis

**Figure 5:**
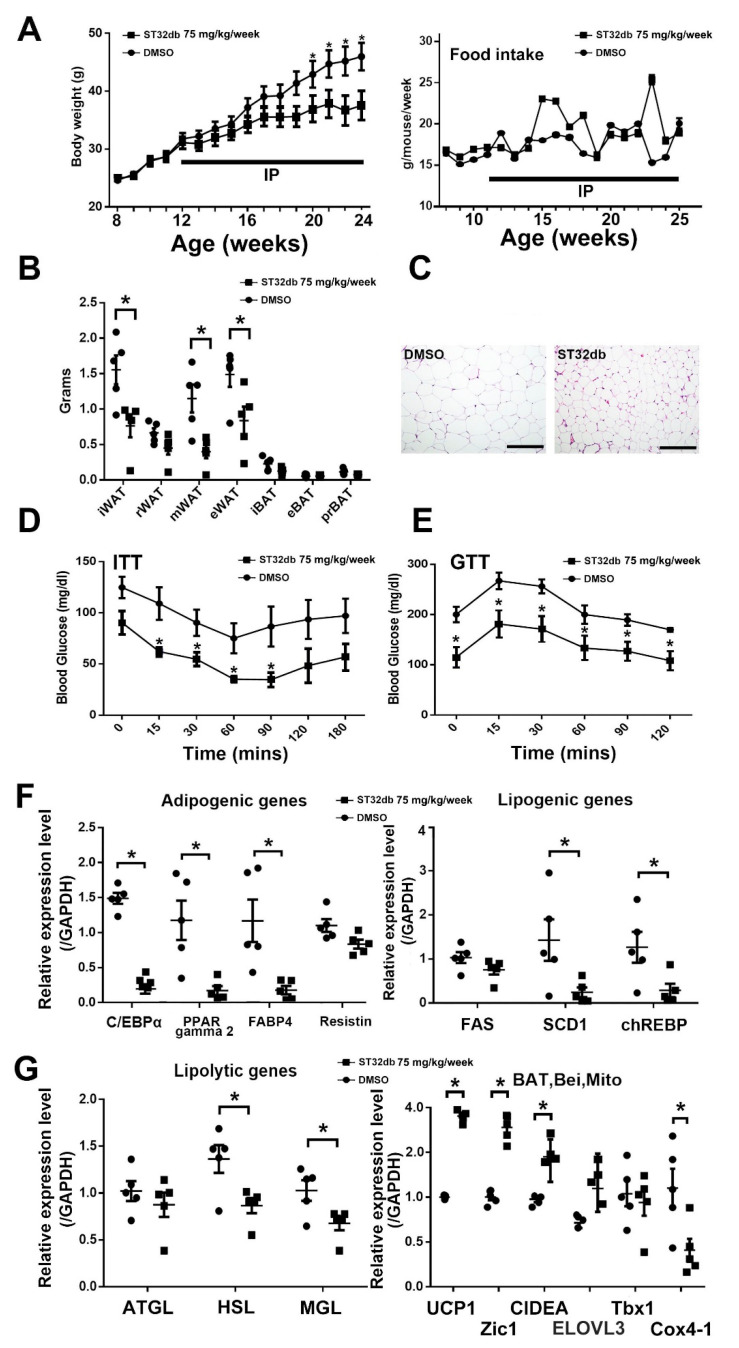
The ATF3 inducer ST32db prevents HFD-induced obesity and metabolic dysfunction. WT mice fed with an HFD for 12 weeks with or without intraperitoneal (i.p.) injection of ST32db at 75 mg/kg/week were analyzed. (**A**) Body weight and food intake. (**B**) Change in depot weight of BAT and WAT. (**C**) H&E staining results for iWAT with and without treatment. (**D**) Insulin tolerance test (ITT) results. (**E**) Glucose tolerance test (GTT) results. (**F**) Real-time PCR analysis results for mRNA levels of adipogenic, lipogenic, and lipolytic genes in iWAT. (**G**) Real-time PCR analysis results for mRNA levels of brown (BAT), beige (Bei), and mitochondria (Mito) genes in iWAT. Data are the mean ± SEM (*n* = 5 per group); * *p* < 0.05 compared with control (DMSO). Scale bar = 100 μm.

To verify the ability of ST32db and ST32C to upregulate ATF3, we used stable clones of 3T3-L1 adipocytes containing constitutively overexpressing ATF3 promoter-luciferase constructs (pGL4.17-ATF3) or pGL4.17 vector as the control. Both ST32db and ST32C increased luciferase activity compared with the control (data not shown). First, we compared the anti-obesity effects of ST32db and ST32C. At the eighth week of ST32C injection at 75 mg/kg/week, the mice died one after another, and we experimented by reducing the dose to 45 mg/kg/week. Nevertheless, as shown in Figure 5A and Figure S3, compared with low-dose ST32C, ST32db suppressed obesity to a great extent, and food intake to a lesser degree in the HFD-fed mice.

Subsequently, we investigated whether ST32db could affect the lipogenesis/lipolysis balance or WAT browning in vivo. Accordingly, after consumption of an HFD for 4 weeks, the HFD-fed obese mice received i.p. injections of ST32db at 75 mg/kg/week or the vehicle, and their food consumption and body weight were recorded weekly. The ST32db-treated mice exhibited reduced body weight compared with the untreated HFD-fed mice, but the difference in food intake was not significant between the two groups (Figure 5A). SME can reduce WAT weight (Figure 1F); therefore, we investigated whether the role of ST32db in mitigating weight gain could be determined by WAT weight reduction. The weights of iWAT, epididymal WAT (eWAT), and mesenteric WAT (mWAT) were reduced in ST32db-treated mice after 12 weeks of treatment compared with untreated mice; however, the weight of retroperitoneal WAT and BAT did not differ between the two groups (Figure 5B). The results observed for changes in adipocyte size were consistent with those observed for changes in adipocyte weight, indicating a decrease in iWAT weight after ST32db treatment (Figure 5C). In addition, ST32db treatment enhanced insulin sensitivity and glucose tolerance in HFD-fed obese mice (Figure 5D,E). Similarly, ST32db treatment decreased adipogenesis/lipogenesis and lipolytic gene expression in the iWAT of HFD-fed mice (Figure 5F,G). Furthermore, ST32db treatment increased the expression of BAT- and beige-related genes (Figure 5G).

**Figure 6:**
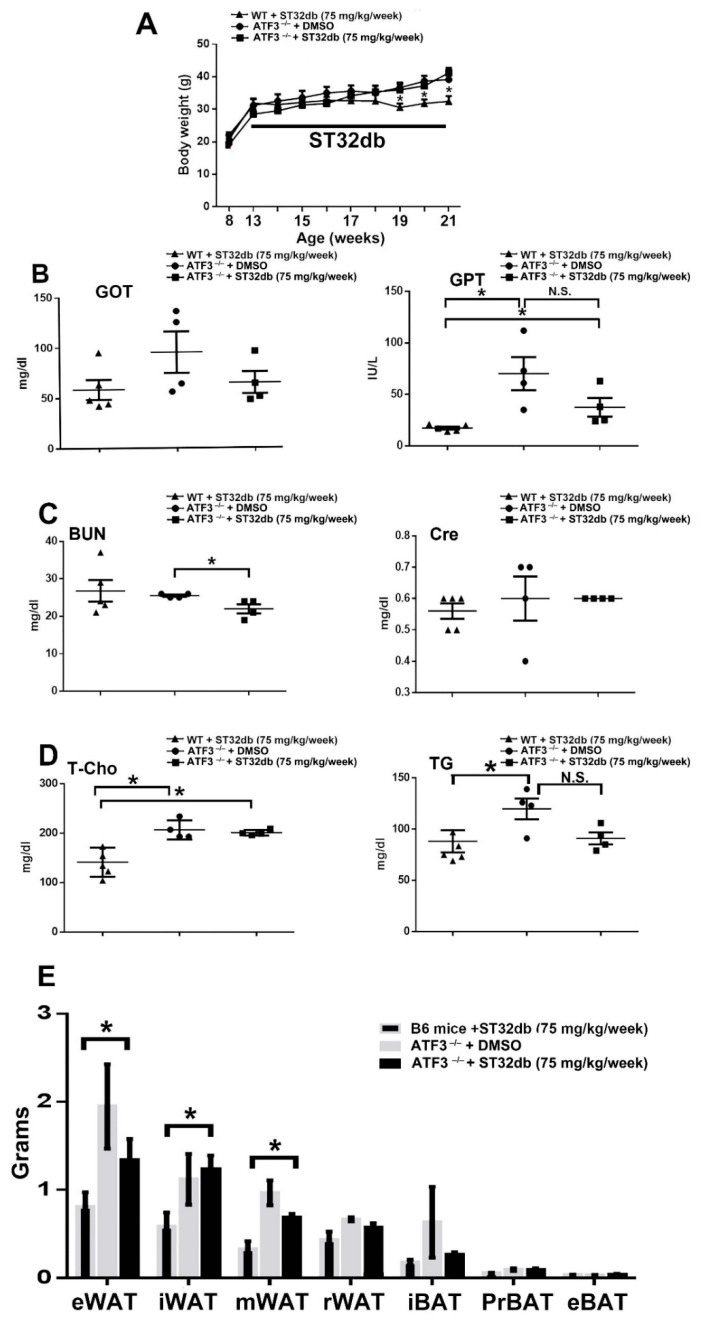
ST32db protects against HFD-induced obesity through an ATF3-specific pathway. ATF3^−/−^ mice fed an HFD for 12 weeks with or without i.p. injection of ST32db at 75 mg/kg/week were analyzed. (**A**) Body weight. (**B**) Serum levels of GOT and GPT. (**C**) Serum levels of BUN and creatinine. (**D**) Serum levels of total cholesterol (T-cho) and triglyceride (TG). (**E**) Weight of epididymal WAT (eWAT), inguinal WAT (iWAT), mesenteric WAT (mWAT), retroperitoneal WAT (rWAT), interscapular BAT (iBAT), peri-renal BAT (prBAT), and epididymal BAT (eBAT). Data are the mean ± SEM; * *p* < 0.05. For (**A**–**E**), *n* = 4 per group, except for WT+ST32db (*n* = 5).

To further verify our hypothesis that ST32db exerts its beneficial effects through ATF3 signaling, HFD-fed ATF3^−/−^ mice with or without ST32db injection were compared with WT mice receiving ST32db injection as the positive control group. We observed that ST32db administration did not reduce the body weight of HFD-fed ATF3^−/−^ mice compared with the placebo group of ATF3^−/−^ mice (Figure 6A). The beneficial effects of ST32db in reducing iWAT, eWAT, and mWAT depot weight were not observed in the HFD-fed ATF3^−/−^ mice (Figure 6B–E). Therefore, ST32db may exert its beneficial effects by activating ATF3, effectively ameliorating obesity and related metabolic disorders in HFD-induced obese mice.

### 3.6. ATF3 Regulates Adipogenesis through ATF3-Mediated Pathways

**Figure 7:**
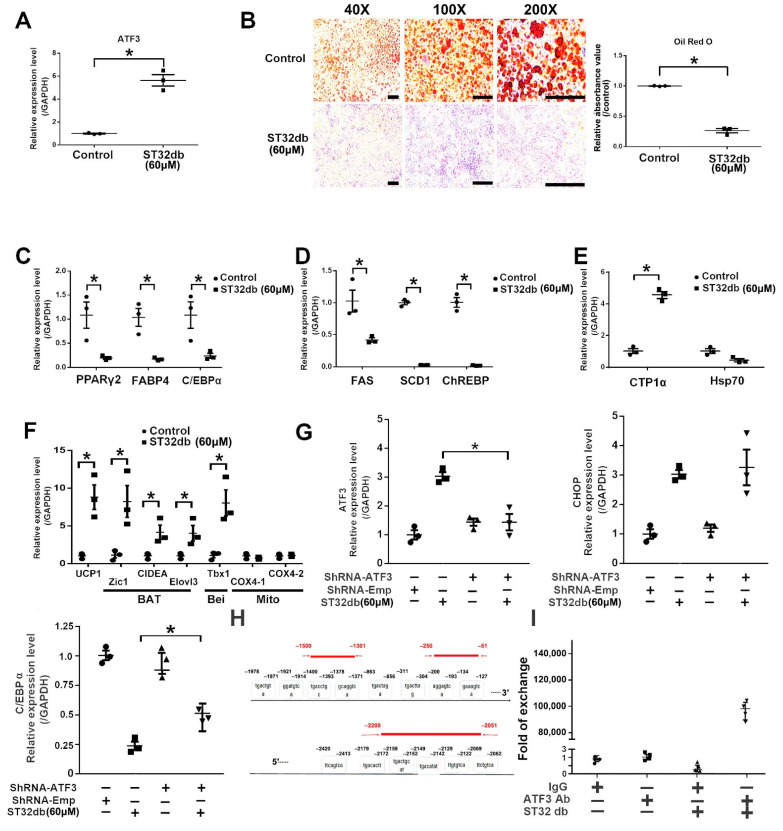
ST32db treatment of 3T3-L1 adipocytes suppresses lipogenesis/adipogenesis through the ATF3 pathway to directly inhibit C/EBPα expression and indirectly inhibit the CHOP pathway. (**A**) ATF3 expression after ST32db treatment. (**B**) Representative photographs and statistical results of Oil Red O staining of 3T3-L1 cells with and without ST32db treatment. (**C**–**F**) Real-time PCR analysis results of mRNA levels of adipogenic, lipogenic, β-oxidation (β-oxi), brown (BAT), beige (Bei), and mitochondrial (Mito) genes, normalized to GAPDH in 3T3-L1 cells with and without ST32db treatment. (**G**) Expression levels of ATF3, CHOP, and C/EBPα in 3T3-L1 cells after 6 h of ST32db treatment. (**H**) Sequence of three potential binding sites for ATF3 in C/EBPα promoter: site 1 (−2200/−2051), site 2 (−1500/−1301), and site 3 (−250/−51) of C/EBPα locus. (**I**) Results of ChIP experiments with ATF3-specific antibody and primers to amplify region site 2 of C/EBPα locus, which contained one predicted ATF3 binding site in 3T3-L1 preadipocytes. Experiments were conducted in triplicate. Data are the mean ± SEM (*n* = 3); * *p* < 0.05. Scale bar for B: Each line segment is equal to 100 μm.

Our preliminary results confirm that SME treatment reduces body weight mainly through early adipogenesis/lipogenesis reduction (Figure 1A). Notably, ST32db, an ATF3 inducer, increased the browning efficiency in WAT in addition to reducing adipogenesis/lipogenesis (Figure 5G). ATF3 expression could be induced by treatment with 60 μM ST32db, which reduced lipid accumulation in 3T3-L1 adipocytes (Figure 7A,B). Furthermore, ST32db-treated cells exhibited reduced adipogenesis and lipogenesis along with increased β-oxidation levels, as well as BAT-related, beige-related, and mitochondrial gene expression (Figure 7C–F). Therefore, the effects of ST32db in 3T3-L1 adipocytes were similar to those of infusion treatment in mice.

A previous study suggested that C/EBPα is a key activator of adipogenesis [38]. To elucidate the molecular mechanism underlying the repression of adipogenesis and inhibition of C/EBPα by ATF3 signaling in white adipocytes, we first transfected 3T3-L1 adipocytes with shRNA-ATF3 to inhibit ATF3 expression following treatment with ST32db; in addition, C/EBPα and CHOP levels were assayed by qPCR. As displayed in Figure 7G, only C/EBPα expression, and not CHOP gene expression, was inhibited by transfection with shRNA-ATF3 after ST32db treatment in 3T3-L1 adipocytes. This indicates that ST32db can directly induce ATF3 to inhibit C/EBPα expression, whereas CHOP is not induced by ATF3. Subsequently, we examined the proximal promoter regions of C/EBPα at the 5′ end, and the analysis results revealed three potential ATF3 binding sites on the C/EBPα promoter (Figure 7H). To confirm this finding, we conducted a ChIP assay with qPCR to examine whether ATF3 could bind to the proximal promoter regions of C/EBPα at its potential binding sites. As shown in Figure 7I, ATF3 could bind to site 2 (−1500 to −1301) but not to site 1 (−250 to −51) or site 3 (−2051 to −2200). Thus, ST32db not only represses adipogenesis/lipogenesis, but also promotes adipocyte transdifferentiation through the ATF3-dependent pathway, directly inhibiting C/EBPα and indirectly inhibiting the CHOP pathway.

## 4. Discussion

Thus far, >50 water-soluble and >30 liposoluble molecules have been isolated from *S. miltiorrhiza* [25]. Among these single-component molecules, water-soluble molecules such as salvianolic acid A and B and magnesium lithospermate B (MLB) and liposoluble molecules such as tanshinone I, danshensu bingpian zhi (DBZ), and tanshinone ⅡA have been reported to exhibit anti-obesity effects [21,28,29,39,40] after approximately 5–10 weeks of treatment in HFD-fed animals. However, in this study, after pretreating mice with an HFD for 4 weeks, we observed that significant body weight reduction was achieved within only 2 weeks after SME injection, and this effect was more significant than after treatment with a single compound (Figure 1). A possible reason for this finding is that the anti-obesity effect of SME is generated by the synergistic action of multiple single molecules. Long-term SME treatment was determined to have relatively fewer toxic effects based on the observed urea/creatinine levels and improved liver function. Of note, when we suspended SME treatment for 1 month and then restarted it for 3 weeks, the body weight of mice decreased again, indicating that SME treatment remains effective in the case of obesity recurrence. These results suggest that SME is ideal for clinical applications and as a health food.

ST32db suppressed adipocyte adipogenesis and promoted the expression of BAT- and beige-related genes (Figure 5); these findings are consistent with some of the recent findings by Ku et al. [21]. They examined the regulatory role of ST32db in the β3-adrenoceptor (β3-AR)/protein kinase A(PKA)/p38 pathway and tried to explain the anti-obesity effect of ST32db in terms of this pathway, because a prior study pointed out that it is an important mechanism for the induction of UCP1 in WAT browning [41]. Nevertheless, their evidence is contradictory and inconsistent. First, their study showed that in 3T3-L1 preadipocytes, ST32db upregulated the β3-AR/PKA/p38 pathway, but that this pathway was downregulated in D16 adipocytes [21]. However, in their animal experiments, ST32db could not effectively upregulate β3-AR or PKA, but downregulated p38 [21]. In addition, they observed that ST32db regulated genes related to adipogenesis, lipolysis, and browning, and these processes were mostly inconsistent between cell and animal experiments. Notably, one consistent finding is that the upregulation of ATF3 by ST32db could be completely reproduced in the three sets of experiments [21]. Our finding is also consistent with the findings of Ku et al. (Figure 7A), implying that the effect of ST32db against obesity is related to ATF3 upregulation. The extant literature suggests that ATF3 is a functionally important downstream effector of p38 [42]. Therefore, if ST32db does indeed exert an anti-obesity effect through the p38 pathway, as proposed by Ku et al., it should promote upregulation of the p38 pathway and then induce the upregulation of ATF3, rather than downregulation, as seen in the animal experiments by Ku et al. [21]. Therefore, Ku et al.’s attempt to use the β3-AR/PKA/p38 or p38-related pathways to explain the anti-obesity effect of ST32db is questionable, and further verification of such a role is required.

Moreover, in the mouse experiment performed by Ku et al., expression of the brown-adipocyte-related biomarkers increased, with no differences in the expression adipogenesis and lipogenesis genes such as C/EBPα, PPARγ, and SCD1 [21], but in our results, their expression was obviously inhibited (Figure 5F). This may be due to the material used by Ku et al., ST32db and ST32da, which were suggested to promote adipocyte browning by Lin et al. [19], are similar in structure. The two compounds are mixed during synthesis, requiring multiple elaborate isolation steps to purify them separately. At the same time, the final synthetic product also needs to be verified by various methods. However, Ku’s study did not perform validation by mass spectrometry and HPLC, as was conducted in our study (Supplementary Figure S2). Therefore, we think that the so-called ST32db compound in Ku et al.’s study may have been a mixture with ST32da as the main component and ST32db as a minor component. In addition, Ku et al. found that ST32db induced browning, leading to weight loss; the dose they used was about 38 times lower than the dose we used (2 vs. 75 mg/kg/week) [21]. This discrepancy is also consistent with the use of an impure compound by Ku et al., because the anti-obesity dose of ST32da (10 mg/kg/week) was previously published by Lin et al. [19], which is substantially different from the ST32db dose (75 mg/kg/week) used in this study. Such a large difference is also consistent with our speculation that the effect of browning and weight loss caused by so-called ST32db (2 mg/kg/week) in Ku et al.’s study was actually due to a mixed effect of ST32da and ST32db.

Based on our previous studies showing that ATF3 overexpression could suppress obesity [19], we focused on ATF3 in the present study. We synthesized two modified components, ST32db and ST32C, which are structurally similar to tanshinone I and IIA. Both ST32db and ST32C can upregulate ATF3; however, their anti-obesity effects are weaker than those of tanshinone I and II, whose effects can be sustained for approximately 5 to 6 weeks after treatment. Nevertheless, in addition to inhibiting obesity, ST32db can ameliorate other obesity-induced metabolic syndromes, such as diabetes (Figure 5D,E), and even diabetic nephropathy (data not shown). Thus, ST32db might be more advantageous than other published single components of *S. miltiorrhiza* for treating obesity and its related metabolic disorders. An analysis of the toxicity of the two single components synthesized in this study demonstrates that the ST32C infusion at 75 mg/kg/week caused the death of mice after 8 weeks of treatment, and the dosage was therefore reduced to 45 mg/kg/week (Figure S3). Nevertheless, ST32C must be administered for approximately 14 weeks before body weight reduction is observed, even without pretreatment with an HFD, although the corresponding side effect would be appetite suppression (Figure S3). ST32C is more toxic than ST32db, possibly due to the extra methyl group in its structure. However, further research must be conducted to determine the actual reason for ST32C toxicity.

C/EBPα and PPARγ are considered to be master regulators of adipogenesis and were found to be critical for in vitro and in vivo adipogenesis [43,44]. C/EBPα is expressed and regulated in the early stages of 3T3-L1 adipocyte differentiation to maintain PPARγ expression [45,46,47]. It has been reported that the overexpression of ATF3 by treatment with lentivirus-mediated [19,22] and ATF3 inducers such as thapsigargin [22] and sulfuretin [23] decreased C/EBPα transcripts in 3T3-L1 cells. These results are consistent with the results in this study showing that SME and ST32db can also induce ATF3 and further inhibit C/EBPα (Figure 2A and Figure 7A,C). Thus, we believe that ATF3 regulates adipogenesis upstream of C/EBPα via transcriptional interference or epigenetic regulation. Results of both previous research [22] and in this paper (Figure 7I) demonstrate, by CHIP assay, that ATF3 can directly bind to the C/EBPα promoter. Nonetheless, we do not rule out the possibility that ATF3 exerts its effects via epigenetic regulation. It has been demonstrated that HDAC1 interacts with C/EBPα and is recruited to the C/EBPα promoter in preadipocytes, thus repressing C/EBPα transcription. When HDAC1 is downregulated by ubiquitination, C/EBPβ is free to interact with acetyltransferase GCN5/p300, resulting in C/EBPα transcription [48]. Our previous study on the epigenetic regulation between ATF3 and HDAC1 indicated that ATF3 could recruit HDAC1 and deacetylated histones, resulting in a condensed chromatin structure, interference with NF-κB binding, and inhibition of inflammatory gene IL6/IL12 transcription activity [49]. In combination with these studies, we rationally speculate that ATF3 in adipocytes may also recruit HDAC1 to the promoter region of C/EBPα to inhibit the level of transcriptional C/EBPα expression.

Interestingly, obesity is characterized by excess fat accumulation in white adipose tissue, in which inflammation is activated through the secretion of pro-inflammatory factors such as IL-6, IL-12, and TNFα, and transcription is activated via the NF-κB activation pathway [50,51]. Following the association between ATF3 and HDAC1, whether obesity is also reduced through the NF-κB pathway and adipogenesis/adipogenesis-targeted genes are regulated by NF-κB will be assessed in future work. As for ATF3-associated proteins other than HDAC1, computational tools have been used to analyze a comprehensive set of transcriptomic data derived from Toll-like-receptor (TLR)-activated macrophages to identify a prominent group of genes, such as those of the Fos and Jun family, which appear to be regulated by ATF3 [52]. Therefore, our next step would be to also explore whether ATF3 associates with c-jun as a heterodimer to induce the expression of genes related to lipolysis and browning. It is suggested in the literature that CHOP negatively regulates adipocyte differentiation [53]. SME and ST32db can upregulate CHOP, although ATF3 shRNA treatment failed to inhibit CHOP upregulation, indicating that ST32db does not cause the subsequent transcriptional upregulation of CHOP via ATF3 upregulation. Therefore, if ST32db can inhibit adipogenesis via a CHOP-related pathway and is a specific inducer of ATF3, this suggests that ATF3 and CHOP should be co-factors interacting with each other at the protein level. Indeed, findings in the literature have shown that ATF3 can form a heterodimer with CHOP, an interacting protein, and regulate downstream genes at the protein level [54]. However, this hypothesis still needs further experimental confirmation.

Although SME and ST32db were observed to have excellent anti-obesity effects in this study, both in vitro and in vivo, this study still has limitations. Our animal study design was based on feeding C57BL/6J mice a high-fat diet. Previous research noted that different mouse strains have different degrees of obesity in response to an HFD; for example, adult 129S mice are more resistant to an HFD than B6 mice [33]. This phenomenon implies that the genome of C57BL/6J mice may be susceptible to an HFD, causing obesity. Of note, clinically, we have observed that many patients develop obesity by eating too much, rather than by eating too much high-fat food. Therefore, our experimental design may be comparable to obesity in only a small subset of people. However, the use of a corresponding animal model, such as leptin-deficient ob/ob mice, could make up for the deficiencies in our experimental design. This rodent model developed hyperphagia, resulting in weight gain and obesity without a high-fat diet [55]. In the future, further experiments should be conducted with SME and ST32db to explore obesity interventions in ob/ob mice.

Today, treatment of obesity and obesity-related metabolic problems remains an unmet clinical need. Previous studies have suggested that ATF3 could be a target for obesity treatment. The results for SME and ST32db in this paper are a proof of concept showing that the prevention of obesity and related metabolic problems can be regulated through ATF3-related pathways. Therefore, from a clinical point of view, the ATF3 inducers ST32db and SME may be ideal weight-loss drugs or herbal health foods. In the future, the transcriptional and epigenetic regulation of ATF3 in adipogenesis represents a new direction for obesity research.

## Data Availability

The data presented in this study are available in the article and the supplementary material.

## Supplementary Materials

The following supporting information can be downloaded from https://www.mdpi.com/article/10.3390/cells11061022/s1: Figure S1. Physiological effects of SME treatment on mouse livers and kidneys; Figure S2. Characteristics of the investigated compounds, ST32db and ST32C, determined by mass spectrophotometry, HPLC, and NMR NOESY; Figure S3. Body weight and food intake of HFD-fed mice injected with ST32C. Table S1. Primer sequences.
