# Supplementary material for: Salvia miltiorrhiza Extract and Individual Synthesized Component Derivatives Induce Activating-Transcription-Factor-3-Mediated Anti-Obesity Effects and Attenuate Obesity-Induced Metabolic Disorder by Suppressing C/EBPα in High-Fat-Induced Obese Mice
Source: Cells. 2022 Mar 17;11(6):1022. doi: 10.3390/cells11061022 (PMC8947163; doi:10.3390/cells11061022)
Supplement: Supplementary file 1 [file cells-11-01022-s001.zip › cells-1596926-supplementary-proof done - Copy/Table S1 Primers.pdf]

Table S1. Primers

mouse primers for Real-time PCR		
**Gene name**	**Forward Primer 5’-3’**	**Reverse Primer 5’-3’**
ATF3	5’CTCCTgggTCACTggTATTTg3’(21)	5’CCgATggCAgAggTgTTTAT3’(20)
C/EBPα	5’TggACAAgAACAgCAACgAgTAC3’(23)	5’gCAgTTgCCCATggCCTTgAC3’(21)
PPARγ2	5’TggAgCCTAAgTTTgAgTTTgC3’(22)	5’CAggTTgTCTTggATgTCCTC3’(21)
FABP4	5’gCTCCTCCTCgAAggTTTAC3’(20)	5’CCCACTCCCACTTCTTTCAT3’(20)
Resistin	5’CTAAgCTgAgggTCTggAAATg3’(22)	5’CACACACCCTTCTCCACTAAAg3’(22)
FAS	5’AgACCCgAACTCCAAgTTATTC3’(22)	5’gCAgCTCCTTgTATACTTCTCC3’(22)
SCD1	5’ggTggAAgAAgTgAgCgAATAg3’(22)	5’gCgTgggCAggATgAAg3’(17)
ChREBP	5’TgTTCAgCATCCTCATCCgACCTT3’(24)	5’TgAgTTggCgAAgggAATTCAggA3’(24)
ATGL	5’TAgCTAACAgTTgggCTTCAC3’(21)	5’CAgAgAgAACAgAgCAgCTTAC3’(22)
HSL	5’CATCAACCACTgTgAgggTAAg3’(22)	5’AAgggAggTgAgATggTAACT3’(21)
MGL	5’gACAgAAAgAgTgTgggAAgAg3’(22)	5’CTgAgCACAgTAgTCTggAATg3’(22)
Cpt1α	5’gAAgTgTCggCAgACCTATTT3’(21)	5’gTCCTCCTCTCTATATCCCTgTT3’(23)
HSP70	5’TggTgCTgACgAAgATgAAg3’(20)	5’CgCTgAgAgTCgTTgAggTAg3’(21)
UCP1	5’gAggTCgTgAAggTCAgAATg3’(21)	5’AAgCTTTCTgTggTggCTATAA3’(22)
Zic1	5’ATATgCgCCAACCCATCAA3’(19)	5’TCgTgCATggTgCTgAAA3’(18)
CIDEA	5’gCAACCAAAgAAATCgggAATAg3’(23)	5’CTCgTACATCgTggCTTTgA3’(20)
Elovl3	5’TACATCTggAggCAggAgAA3’(20)	5’ggTggAAgAAgTgAgCgAATAg3’(22)
Tbx1	5’CCCgATTCCATgTTgTCTATgT3’(22)	5’gTgAAgCgTgTCTCCTCAAA3’(20)
Cox4-1	5’AgTTgTACCgCATCCAgTTT3’(20)	5’gCAgTgAAgCCAATgAAgAAC3’(21)
Cox4-2	5’ACgAATggAAgACAgTgATgg3’(21)	5’gTCAgggTgACAACCTTCTTAg3’(22)
CHOP	5’CCCAggAAACgAAgAggAAg3’(20)	5’gAACTCTgACTggAATCTggAg3’(22)
GAPDH	5’ggAgCCAAACgggTCATCATCTC3’(23)	5’gAggggCCATCCACAgTCTTCT3’(22)

**mouse** Primers for ChIP assay.		
CEBPα promoter	Forward Primer 5’-3’	Reverse Primer 5’-3’
Primer 1	5’CgCCgCCggggTggggCTgAgC3’	5’TggTgTCCAAACgggTCTCggA3’
Primer 2	5’CAgCAgACCTCCCTAACAgTTT3’	5’gCACCCCgAAgACCgCggCTTC3’
Primer 3	5’CTTTggTgTCCCCCTCTATgAT3’	5’TCTCCAgTCgCTgACAgAATCC3’
