# Supplementary figures and images for: Salvia miltiorrhiza Extract and Individual Synthesized Component Derivatives Induce Activating-Transcription-Factor-3-Mediated Anti-Obesity Effects and Attenuate Obesity-Induced Metabolic Disorder by Suppressing C/EBPα in High-Fat-Induced Obese Mice
Source: Cells. 2022 Mar 17;11(6):1022. doi: 10.3390/cells11061022 (PMC8947163; doi:10.3390/cells11061022)

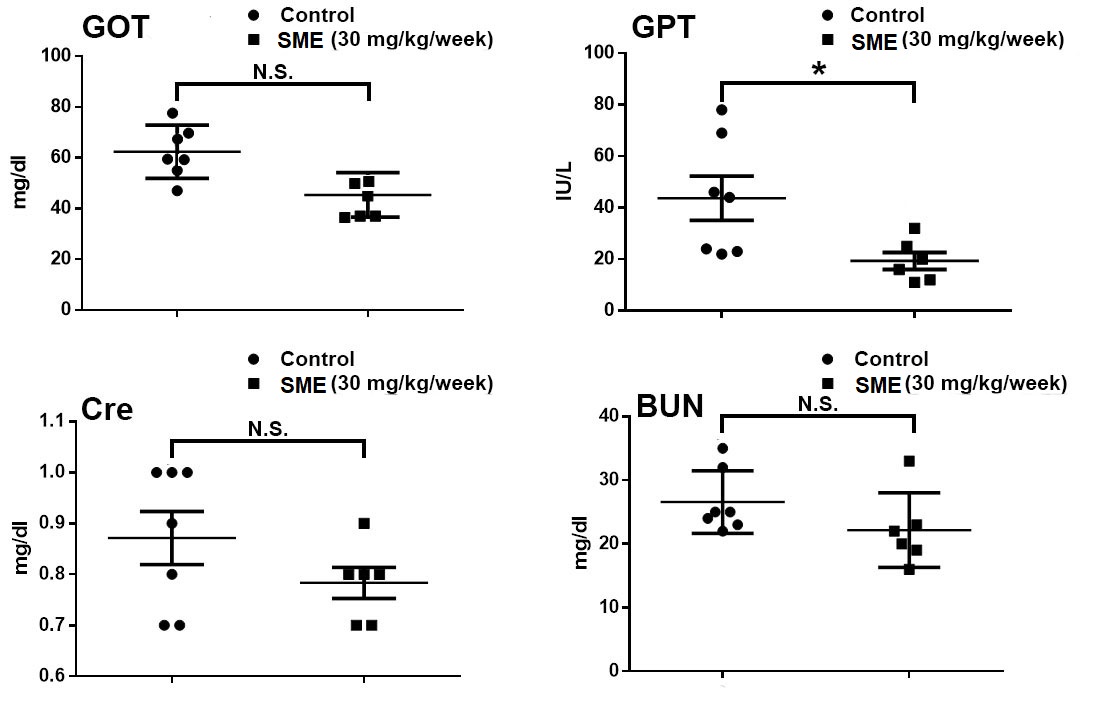

Supplement: Supplementary file 1 [file cells-11-01022-s001.zip › cells-1596926-supplementary-proof done - Copy/sup fig 1.jpg]

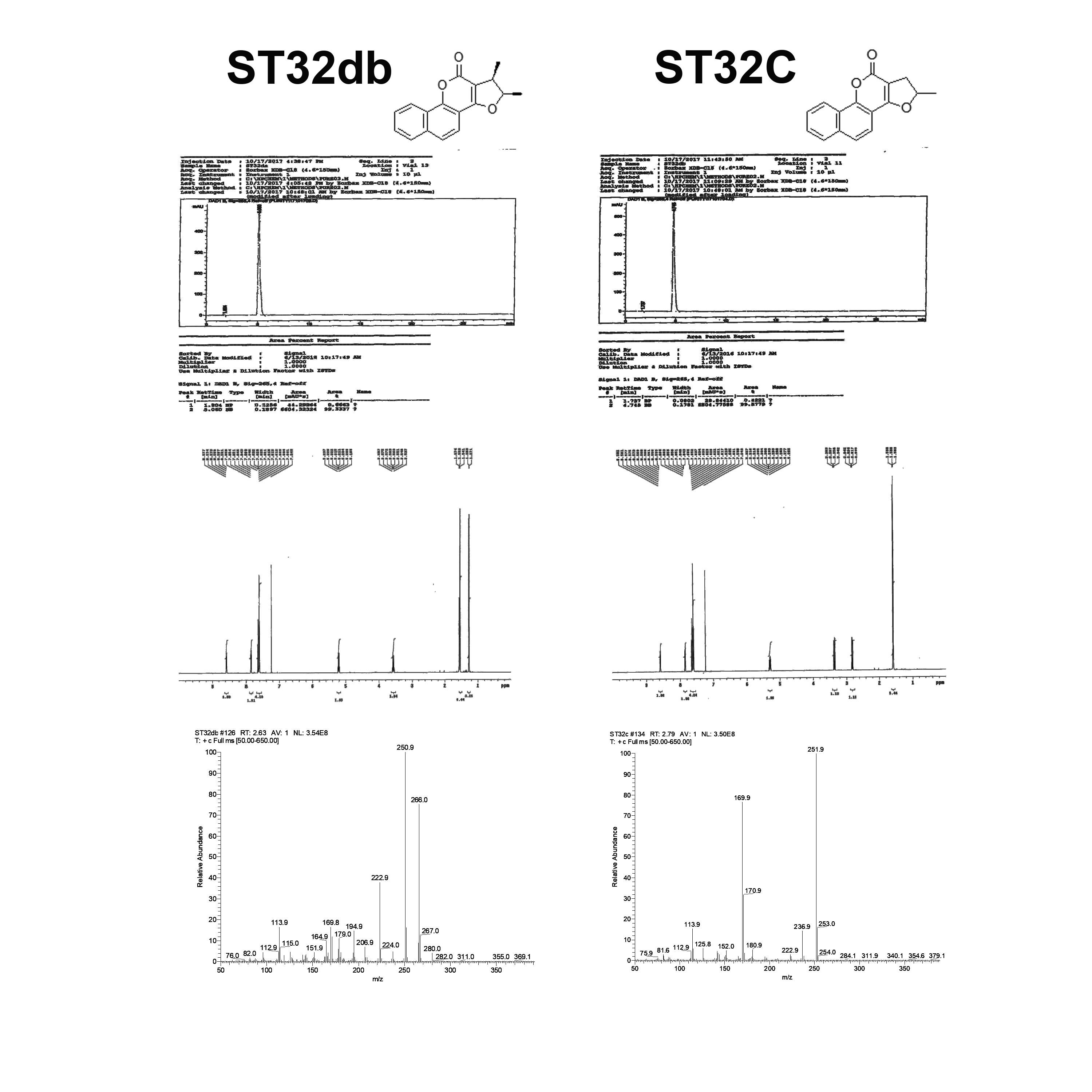

Supplement: Supplementary file 1 [file cells-11-01022-s001.zip › cells-1596926-supplementary-proof done - Copy/supplementary fig 2.jpg]

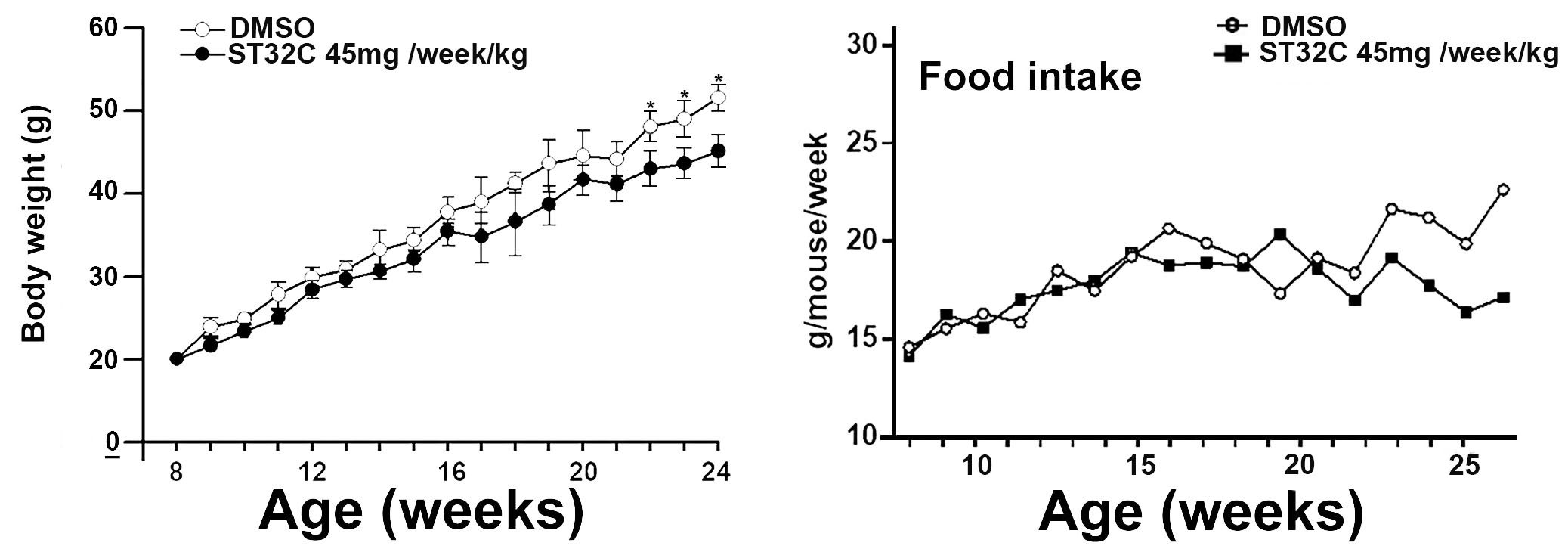

Supplement: Supplementary file 1 [file cells-11-01022-s001.zip › cells-1596926-supplementary-proof done - Copy/supplementary fig 3 +food intake.jpg]
